# Hyperattenuated Lesions on Immediate Non-contrast CT After Endovascular Therapy Predict Intracranial Hemorrhage in Patients With Acute Ischemic Stroke: A Retrospective Propensity Matched Study

**DOI:** 10.3389/fneur.2021.664262

**Published:** 2021-08-05

**Authors:** Nannan Han, Gejuan Zhang, Yige Li, Haojun Ma, Hanming Ge, Xiao Zhang, Yong Zhao, Shilin Li, Leshi Zhang, Yanjun Gao, Wenzhen Shi, Peng Yan, Wu Li, Mingze Chang, Ye Tian

**Affiliations:** ^1^Department of Neurology, The Affiliated Hospital of Northwest University, Xi'an No.3 Hospital, Xi'an, China; ^2^Xi'an Key Laboratory of Cardiovascular and Cerebrovascular Diseases, The Affiliated Hospital of Northwest University, Xi'an No.3 Hospital, Xi'an, China; ^3^Precision Health Institution, GE Healthcare China, Shanghai, China; ^4^Medical Research Center, The Affiliated Hospital of Northwest University, Xi'an No.3 Hospital, Xi'an, China; ^5^Department of Radiology, The Affiliated Hospital of Northwest University, Xi'an No.3 Hospital, Xi'an, China; ^6^The College of Life Sciences, Northwest University, Xi'an, China

**Keywords:** acute stroke, thrombectomy, NCCT, hemorrhage, hyperattenuated lesions

## Abstract

**Background and Purpose:** This study aimed to analyze the association between hyperattenuated lesions (HALs) and postoperative intracranial hemorrhage (IH) and predict perioperative IH through quantitative analysis of HALs in acute ischemic stroke (AIS) with anterior large vessel occlusion (LVO) after endovascular therapy (ET).

**Materials and Methods:** This retrospective, propensity-matched study enrolled AIS who received ET from a single-center registry study between August 2017 and May 2020. The enrolled patients were divided into two groups: IH and non-IH, by follow-up postoperative CT. The occurrences of HALs on immediate CT after ET were also recorded. The association between IH and HALs after propensity score matching (PSM) was determined by binary logistic regression models. The receiver operating characteristic (ROC) curve was used to determine the predictive value of the highest CT Hounsfield units (HU) value on immediate CT.

**Results:** Initially, 1,418 patients who underwent digital subtraction angiography were reviewed and 114 AIS patients with immediate postoperative CT and follow-up CT after ET were enrolled. Forty-nine out of the 114 patients developed IH after therapy. After PSM analysis, patients with IH were more likely to have HALs on immediate CT (Odds Ratio, OR 11.9, *P* = 0.002, and 95% CI: 2.485–57.284). For 80 patients with HALs, ROC analysis of the highest CT value in the HALs territory showed that the cut-off value was 97 HU, the sensitivity was 70.21%, and the specificity was 81.82%.

**Conclusions:** Patients with HALs after ET are more likely to have perioperative IH. The highest CT value in the HALs area might be used to predict IH.

## Introduction

Endovascular interventional therapy, especially mechanical thrombectomy (MT), has extended the treatment window of acute ischemic stroke (AIS) to 16–24 h (1, 2). MT has become the standard treatment for AIS patients with large vessel occlusion (LVO) (3). Cerebral hemorrhage after interventional therapy is a serious complication (4). Early detection of hemorrhagic transformation after interventional therapy can guide blood pressure control (5), sedative use, and antiplatelet therapy (6). In particular, it is important to evaluate the risk of hemorrhage or hemorrhagic transformation after infarction in patients with mTICI ≤ 2b and patients with emergency stent implantation and balloon dilatation who need antiplatelet to maintain blood flow. Postoperative non-contrast computed tomography (NCCT)/dual-source CT is used as a routine examination after thrombectomy to distinguish whether there is contrast agent extravasation or hemorrhage (7). Dual-energy CT has superior performance in terms of differentiating contrast agent extravasation from hemorrhage. However, NCCT scans are obtained in centers where dual energy scans are not available. At present, some retrospective cohort studies suggest that the immediate postoperative hyperdensity sign is related to the patient's cerebral hemorrhage (8). However, there is no established quantitative method on postoperative NCCT to predict perioperative intracranial hemorrhage (IH) after endovascular interventional therapy. The purpose of this study is to investigate the association between the HALs and IH after therapy and to perform a quantitative analysis of hyperattenuated lesion (HALs) territory on immediate CT to predict IH after interventional therapy to provide warning of early use of antiplatelet drugs and help perioperative patient management.

## Materials and Methods

### Patients

The patients in this study came from A New Parameter Derived from DSA to Evaluate Anterior Cerebral Perfusion (NCT03607565) of the Department of Neurology of the Xi'an No.3 Hospital and reviewed by the Department of Neurology between August 2017 and May 2020. The inclusion criteria of this study were patients with: (1) cerebral infarction caused by acute intracranial and extracranial anterior circulation large artery occlusion (ICA, MCA M1-M2 segment); (2) endovascular interventional therapy (thrombus aspiration, mechanical thrombus removal, emergency balloon dilation, and emergency stent implantation) was performed; (3) Immediate head CT were performed after intra-arterial therapy; (4) Follow-up CT was performed 2–7 days after endovascular treatment. The exclusion criteria were patients with (1) Head CT with hemorrhagic signs on admission; (2) Vertebral-basal artery infarction; (3) Cerebral infarction caused by blockage of the venous system; (4) Arterial thrombolytic therapy; (5) Incomplete head CT follow-up.

Mechanical thrombectomy and balloon/stenting are different procedures but they both fall into the endovascular treatment (ET) category that removes blood clots. The main mechanism of postoperative hemorrhage transformation is the destruction of the blood-brain barrier. This study is focused on the management of postoperative hemorrhage through the quantitative analysis of HALs on immediate CT but not specific procedures. Therefore, the two groups of patients were not distinguished. This retrospective study was approved by the local Institutional Review Board at the Xi'an No.3 Hospital (No. SYXSLL-2018-010), and the requirement for informed patient consent was waived due to the retrospective nature of this study.

### Data Collection

We used the following baseline characteristics of patients from the database: age, sex, previous stroke, side of stroke, initial stroke severity assessed by the National Institutes of Health Stroke Scale (NIHSS), initial CT examination evaluated by Alberta Stroke Program Early CT Score (CT-ASPECTS), intravenous alteplase, hypertension, diabetes mellitus, atrial fibrillation, smoking, tirofiban, thrombolysis in cerebral infarction (TICI), and operational methods.

### CT Imaging and HALs Measurement

CT image scanning was completed through two items of equipment (SOMATOM Definition Flash SIEMENS and Optima CT 680 GE) randomly and then uploaded to the picture archiving and communication systems (PACS). The first head CT was performed immediately after endovascular interventional therapy and the time from the acquisition of the last image of the DSA treatment to the immediate head CT was 38 ± 15 min. The highest CT value of the HALs territory was recorded. The follow-up CT scan was routinely performed 24 h after endovascular treatment, and: (1) If the patient had no IH on 24 h follow-up CT, the head CT should be terminated. (2) If the patient had IH on the 24 h follow-up CT, the head CT was scanned dynamically from 1–7 days after surgery until the IH became stable. The time from the acquisition of the last image of the DSA treatment to the follow-up head CT was 42 ± 25 h.

HALs are observed on immediate CT after interventional therapy, especially in the area of cerebral infarction. The commercial software that comes with the PACS system was used to draw the areas of high-density signs. Bone structure, choroid, and pineal gland were avoided. The highest CT value in the selected volume represents the most severely damaged area of the blood-brain barrier.

All head CT data were obtained by neuroradiologists with more than 5 years of experience and viewed on PACS. In addition, the IH was confirmed by CT after several follow-ups. Like other neuroimages, all CT images were analyzed separately by a neurologist and a neuroradiologist, and disagreement was resolved by reaching a consensus. If no consensus could be reached, another reviewer made the final decision.

### Endpoints

The primary outcome was the association between HALs in immediate CT and early perioperative IH. The second outcome was a receiver operating characteristic (ROC) curve analysis of the highest CT value in HALs territory in patients with HALs to help predict the perioperative IH. Total IH was defined according to the European Cooperative Acute Stroke Study (ECASS)II trial (9).

### Statistical Analysis

All enrolled patients were divided into two groups: IH and non-IH (control group) groups by follow-up CT after endovascular interventional therapy. Continuous variables were expressed as the mean ± standard deviation or median (inter-quartile range, IQR). Single Student's *t*-test was used to detect the differences between the groups. For categorical variables, frequency and percentage were used to summarize data, and between-group comparisons were performed *via* the Chi-square, Continuity Correction, or Fisher's exact test, as appropriate.

A propensity score matched (PSM) study is a quasi-experimental method in which statistical technique is used to construct an artificial control group. PSM is used to focus on the relationship between IH and HALs in patients whose other clinical characteristics are matched to obtain the OR.

PSM analysis was done using a multivariable logistic regression model based on: age, sex, previous stroke, side of stroke, NIHSS, CT-ASPECT, IV alteplase, hypertension, diabetes mellitus, atrial fibrillation, smoking, tirofiban, TICI score, and operation method. Pairs of patients with IH or non-IH were derived using 1:1 greedy nearest neighbor matching within PS score of 0.2. This strategy resulted in 37 matched pairs in each group. The balance of measured variables between groups after propensity score-matching was analyzed using a paired Student's *t*-test for continuous measures and McNemar test for categorical variables. Differences in the HALs after propensity score-matching were analyzed using binary logistic regression. Odds ratio (OR) was calculated as an estimate of the risk associated with HALs with 95% confidence intervals (CI). All data were analyzed using SPSS 22.0 (IBM, Armonk, NY, USA) with a significance level of *p* < 0.05 (2-sided).

To predict IH from the highest CT value in HALs territory, a ROC curve analysis was used. Optimal cut-off values to predict IH were calculated by Youden index using MedCalc 15.0 software, and *p* < 0.05 (2-sided) was considered statistically significant.

## Results

**Figure 1:**
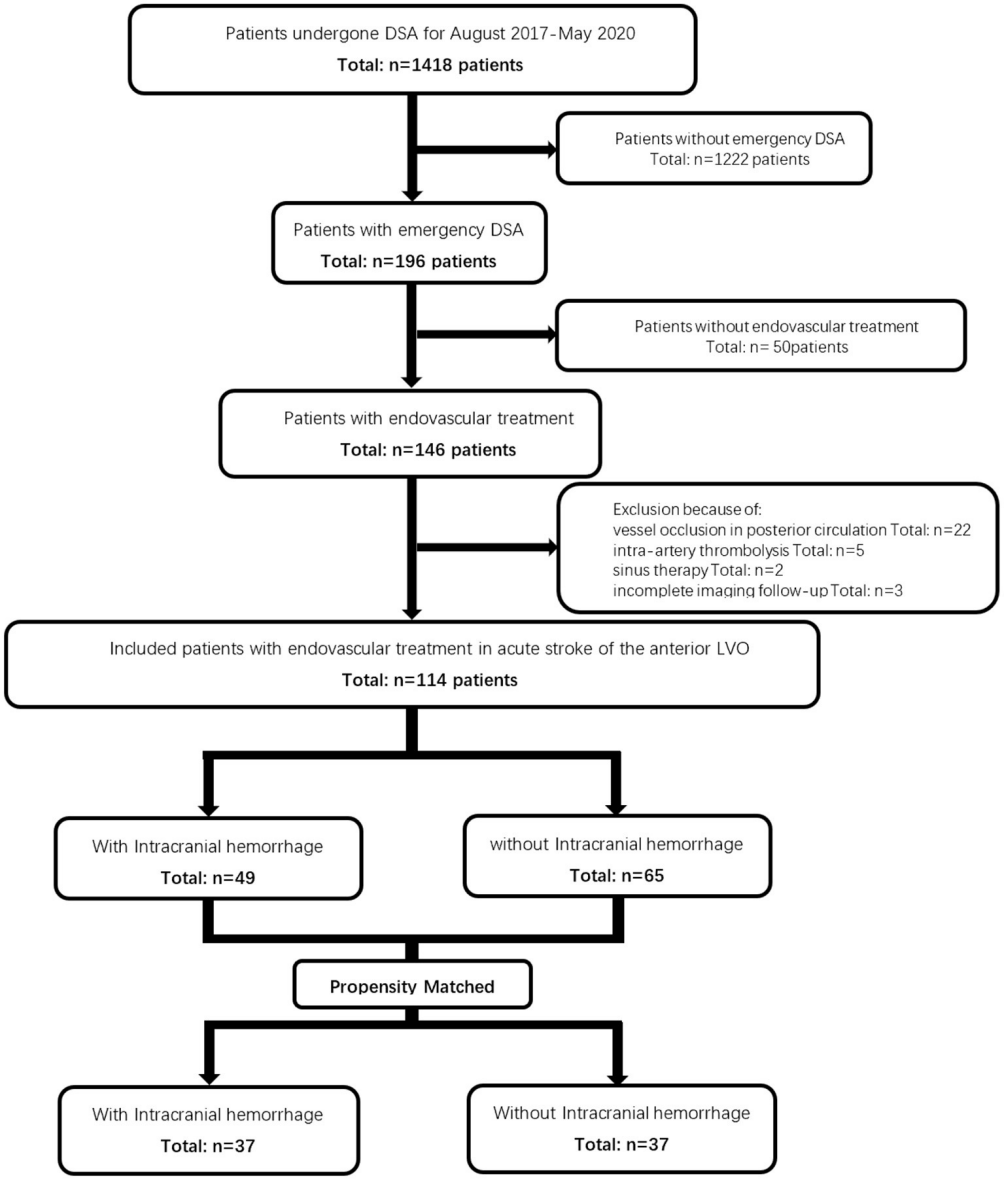
Initially, 1,418 eligible patients were identified from a database. Of those 1,418 patients, 114 patients were enrolled in this study. Among them, 49 patients had an intracranial hemorrhage on follow-up CT while 65 patients did not. In total, 37 matched pairs were identified after the propensity match.

There were 1,418 eligible patients in the database between August 2017 and May 2020. Among the 196 patients with emergency digital subtraction angiography (DSA), 114 met the study-specific inclusion and exclusion criteria and were enrolled for this study. Among the 82 patients excluded, 50 patients were without endovascular therapy, 22 had vessel occlusion in posterior circulation, five had intra-artery thrombolysis, two had sinus therapy, and three had incomplete imaging follow-ups (Figure 1).

Of the 114 patients in the study, 66.7% were male and the mean age was 66.5 ± 13.2. A summary of baseline characteristics of both the entire patients and propensity matched patients (except HALs) is shown in Table 1. Patients with IH tended to have high NIHSS score (*p* = 0.013), low CT-ASPECT score (*P* = 0.049), atrial fibrillation (*p* < 0.001), thrombectomy (*p* < 0.001), and HALs (*p* < 0.001).

**Table 1:** Baseline clinical characteristics according to IH.

**Variables**	**All patients**			**Propensity-matched patients**		
	**IH (*n* = 49)**	**Non-IH (*n* = 65)**	**p**	**IH (*n* = 37)**	**Non-IH (*n* = 37)**	***p***
Age, mean ± SD, year	68.6 ± 13.3	65.0 ± 13.0	0.151	69.3 ± 13.5	67.0 ± 14.2	0.466
Male, *n* (%)	32 (65.3)	44 (67.7)	0.072	24 (64.9)	23 (62.2)	1.00
Previous stroke, *n* (%)	7 (14.3)	17 (26.2)	0.124	7 (18.9)	8 (21.6)	1.00
Side of stroke (right), *n* (%)	26 (53.1)	27 (41.5)	0.222	19 (51.4)	19 (51.4)	1.00
NIHSS, median (IQR)	16 (12, 20)	13 (8,19)	0.013^*^	15 (12,19)	16 (8.5, 20)	0.569
CT-ASPECT, mean ± SD	8.92 ± 1.72	9.46 ± 0.92	0.049^*^	9.22 ± 1.16	9.35 ± 1.09	0.625
IV alteplase, n (%)	19 (38.8)	21 (32.3)	0.474	16 (43.2)	17 (45.9)	1.00
**Vascular risk factors**, ***n*****(%)**						
Hypertension	26 (53.1)	41 (63.1)	0.282	20 (54.1)	25 (67.6)	0.359
Diabetes mellitus	8 (16.3)	15 (23.1)	0.374	6 (16.2)	8 (21.6)	0.754
Atrial fibrillation	30 (61.2)	17 (26.2)	0.000^*^	22 (59.5)	17 (45.9)	0.227
Smoking	13 (26.5)	25 (38.5)	0.181	9 (24.3)	10 (27.0)	1.00
**Procedural details**, ***n*****(%)**						
Balloon/Stenting, *n* (%)	7 (14.3)	30 (46.2)	0.000^*^	6 (16.2)	8 (21.6)	0.754
Tirofiban	18 (36.7)	35 (53.8)	0.070	14 (37.8)	19 (45.9)	0.629
TICI=2b/3	43 (87.8)	61 (93.8)	0.255	31 (91.9)	34 (91.9)	1.00
**HALs**	47 (95.9)	33 (50.8)	0.000^*^	35 (94.6)	22 (59.5)	0.001^*^

*IH, intracranial hemorrhage; SD, standard deviation; IQR, interquartile range; NIHSS, National Institutes of Health Stroke Scale; CT-ASPECT, Alberta Stroke Program Early CT score; IV, intravenous; TICI, Thrombolysis in Cerebral Infarction*.

* *A p < 0.05 indicates statistical significance*.

After performing propensity score matching (except HALs), a total of 37 matched pairs (37 patients from the IH group and 37 patients from the non-IH group) were generated. There were no significant differences in baseline, vascular risk factors and procedural details characteristics for the propensity score matched subjects except for HALs (Table 1). The IH group was associated with HALs than the non-IH group in the matched patients (*p* < 0.001).

A summary of characteristics of the patients after propensity matched according to the IH or non-IH is shown in Table 2. Patients with IH showed association with higher rates of HALs with OR = 11.9, *p* = 0.002, and 95% CI: 2.485–57.284.

**Table 2:** Univariate logistic regression for predicting IH after propensity score matched.

**Variables**	**Univariate**			
	**B**	**OR**	**95%CI**	***p***
Age	0.012	1.012	0.979–1.047	0.478
Sex	−0.117	0.890	0.345–2.294	0.890
Previous stroke	−0.167	0.846	0.272–2.633	0.846
Side of stroke (right)	0.000	1.000	0.402–2.489	1.00
NIHSS	0.021	1.021	0.952–1.095	0.561
CT-ASPECT	−0.110	0.896	0.592–1.354	0.601
IV alteplase	−0.109	0.896	0.358–2.243	0.815
**Vascular risk factors**				
Hypertension	−0.571	0.565	0.220–1.452	0.236
Diabetes mellitus	−0.354	0.702	0.217–2.268	0.554
Atrial fibrillation	0.546	1.725	0.687–4.335	0.246
Smoking	−0.142	0.868	0.305–2.466	0.790
**Procedural details**				
Balloon/Stenting	−0.354	0.702	0.217–2.268	0.554
Tirofiban	−0.334	0.716	0.283–1.810	0.480
TICI = 2b/3	0.000	1.000	0.188–5.309	1.00
**HALs**	2.479	11.932	2.485–57.284	0.002^*^

*OR, odds ratio; CI, confident interval; IH, intracranial hemorrhage; NIHSS, National Institutes of Health Stroke Scale; CT-ASPECT, Alberta Stroke Program Early CT score; IV, intravenous; TICI, Thrombolysis in Cerebral Infarction*.

* *A p < 0.05 indicates statistical significance*.

**Figure 2:**
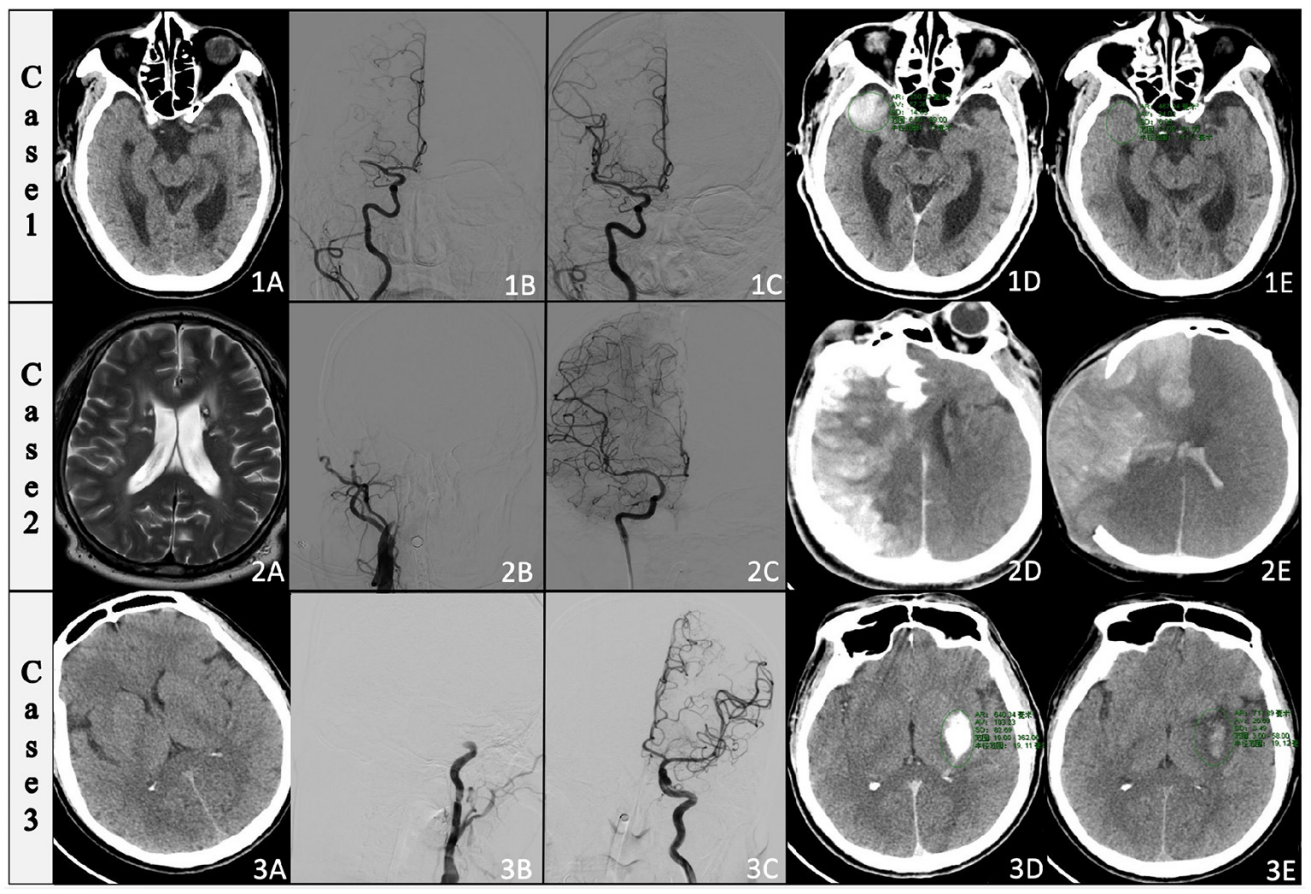
Examples of HALs in patients with acute large vessel occlusion in anterior circulation. CASE-1 demonstrates extravasation of iodine: **(1A)** Head CT before ET, **(1B)** DSA showed right M1-segment occlusion that was successfully recanalized **(1C)**. On immediate CT **(1D)**, there were HALs in the right temporal lobe and the highest CT value was 89 HU. The HALs on follow-up CT performed at 24 h after thrombectomy **(1E)** had vanished. CASE-2 shows IH after thrombectomy: **(2A)** Head CT before ET and DSA demonstrated right proximal internal carotid artery occlusion **(2B)** that was recanalized after endovascular treatment **(2C)**. The immediate CT showed a large amount of HALs in the right hemisphere **(2D)**. We measured the highest CT value in the HALs territory to be 413 HU. The follow-up CT showed hemorrhage in the right cerebral hemisphere on the 3 days **(2E)**. CASE-3 shows minor IH after thrombectomy: **(3A)** Head CT before ET and DSA showed distal occlusion of the internal carotid artery **(3B)** and was successfully recanalized **(3C)**. The immediate CT **(3D)** showed HALs in the left ganglia with the highest CT value of 362 HU. The hemorrhage showed in the follow-up CT **(3E)** was examined at 24 h.

Typical of HALs in patients with acute large vessel occlusion in anterior circulation (Figure 2). CASE-1 demonstrates extravasation of only iodine (1A–E): A patient with right M1-segment occlusion and successfully recanalized. The highest CT value was 89 HU on immediate CT and HALs vanished on follow-up CT. CASE-2 shows IH after thrombectomy (2A–E): a patient with right proximal internal carotid artery occlusion that was reperfusion after endovascular treatment. The immediate CT shows a large amount of HALs in the right hemisphere and the highest CT value in the HALs territory was 413 HU. Follow-up CT shows hemorrhage in the right cerebral hemisphere on the 3 days. CASE-3 shows minor IH after thrombectomy (3A–E): a patient with distal occlusion of the internal carotid artery and successfully recanalized. The immediate CT shows HALs in the left ganglia with the highest CT value was 362 HU and the IH showed in the follow-up CT was examined at 24 h.

**Figure 3:**
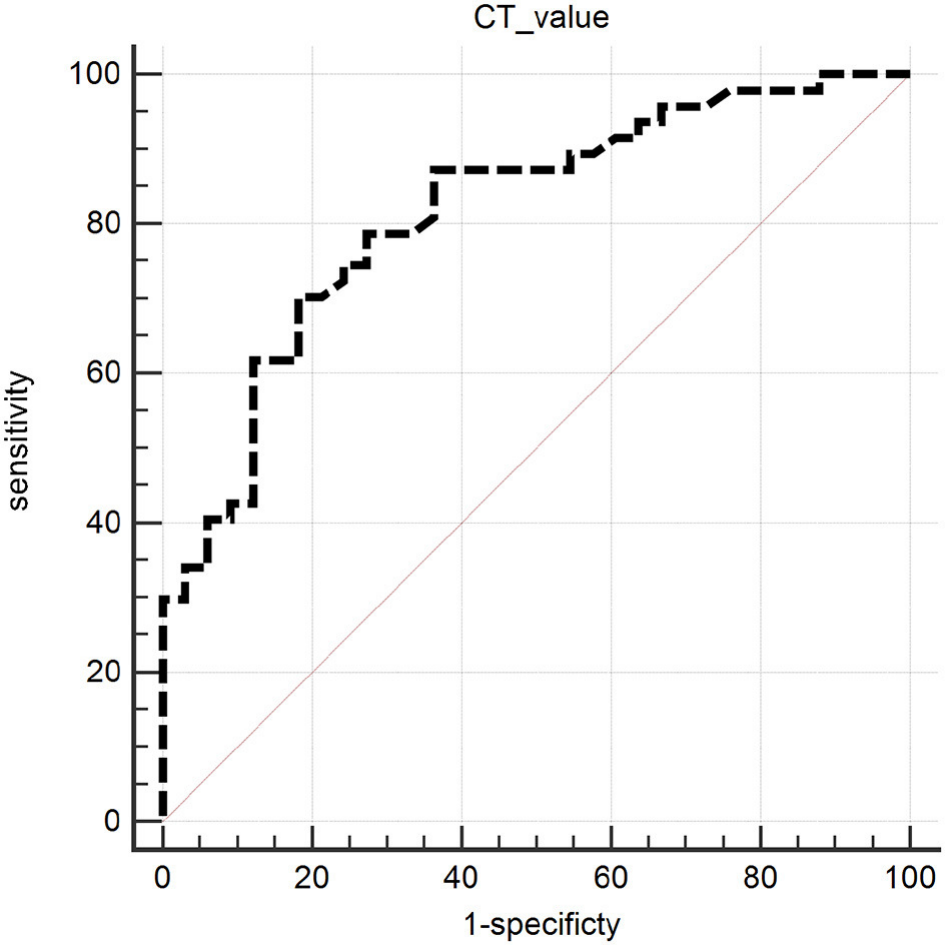
ROC curve analysis of the highest CT value in HALs territory to predict IH. The highest CT value in HALs was a predictive factor for IH with the area under curve (AUC) of 0.816 (95% confidence interval [CI] 0.713–0.894, *p* < 0.001). ROC, receiver-operating characteristic.

A ROC curve analysis was used to evaluate the highest CT value in HALs territory to predict the IH in the perioperative period (Figure 3). An AUC of 0.816 (95% confidence interval [CI] 0.713–0.894, *p* < 0.001) was obtained. A cut-off value of 97 offered the best accuracy in predicting IH with the sensitivity of 70.21%, specificity of 81.82%, positive predictive value (PPV) of 84.62%, and negative predictive value (NPV) of 65.85%.

## Discussion

This present study indicates that, first, the appearance of HALs was closely associated with perioperative IH. Overall, after balancing other baseline characteristics, the risk of having IH in patients with HALs on immediate CT after endovascular treatment was 11.9 times that in patients without. Secondly, in patients with HALs, the highest CT value in the HALs territory might be used to predict the occurrence of perioperative IH, with a sensitivity of 70.21% and a specificity of 81.82% and the cut-off value is 97 HU.

In our study, the probability of IH during the perioperative period was 43.0%, and the probability of HALs on immediate CT after interventional therapy was 70.2%. Previous studies have shown that the probability of postoperative IH fluctuates between 28 and 60.3% (8, 10). The probability of HALs after endovascular therapy fluctuates between 20.8 and 60.8% (8, 10–12), The postoperative HALs in this study are higher than in previous studies. The differences might be explained by: (1) The extravasation of the contrast agent after the operation is mostly metabolized from a few hours to 24 h after operation (11, 12). Therefore, the time of the immediate CT after the operation is relatively important. In this study, the interval between the acquisition of the last image of the DSA and immediate CT was 38 ± 15 min. (2) Patients with thrombus removal in the posterior circulation were excluded in this study because the blood-brain barrier and ischemic tolerance were different than those in the anterior circulation (13).

The risk ratio of IH in patients with HALs was 11.9 times that of patients without after PSM. This data is higher than OR 4.5 (95%CI: 1.22–16.37) in previous reports (10). There are two possible reasons. First, in this study, all types of hemorrhage including symptomatic and non-symptomatic were recorded in follow-up CT. Second, for Chinese patients, intracranial atherosclerotic disease (ICAS) is generally more frequently observed as the cause of LVO than that for patients from the Western world (14). Thus, in such patients, it may have a longer procedure duration and more contrast agent usage. In this study, patients who received balloon dilatation or emergency stent implantation accounted for 32.5% (37/114). Thirdly, randomization was used in this study to eliminate unmatched cases. The reason why reperfusion interval was not included in the analysis in this article is that patients with an onset of more than 4.5 h underwent a mismatch assessment of diffusion-weighted imaging (DWI) and arterial spin labeling (ASL) to identify salvageable brain tissue.

To our knowledge, no previous studies have used the highest CT value on immediate NCCT after endovascular treatment to predict perioperative IH. Some studies have shown that the average HU in HALs territory was significantly higher in the IH group identified by immediate dual energy CT than that in the non-IH group after surgery (377.9 ± 385 HU vs. 83.5 ± 37.9 HU; *p* < 0.0001) (15). The cut-off value of the lesion HU for differentiating IH, which was calculated by ROC analysis, was 80 HU; this value had 100% sensitivity, 63.8% specificity. A hand drawn HALs area lacks repeatability and consistency, thus bringing inaccuracy to the average HU value. The highest CT value represents the severity of the blood-brain barrier damage from stroke blood-brain barrier damage mechanism (16). The appearance of HALs on immediate CT after endovascular therapy might attribute to the following reasons: (1) The extravasation of the contrast agent alone. (2) Contrast-agent-mixed red blood cell extravasation. (3) The rupture of the blood vessel caused a large amount of contrast agent and blood to leak out of the arteries. Ischemia and hypoxia lead to rapid metabolism of glucose reserves, resulting in accumulation of lactic acid in brain tissue, and subsequent changes in cell structure, which in turn leads to the release of pro-inflammatory factors, oxidants, and proteolytic enzymes, which ultimately leads to cell damage and rupture of the blood-brain barrier (16). As the destruction of the blood-brain barrier increases, the exudation of the iodine contrast agent increases (17). When the vascular permeability further increases, the red blood cell mixed with iodine contrast agent leaks out of the blood vessel to cause hemorrhage transformation, and the iodine contrast agent is absorbed and metabolized at the 24 h postoperative follow-up CT (11), while the red blood cells are still retained in the brain tissue of the infarct area. Intraoperative blood vessel rupture caused the mixed blood of the iodine contrast agent to leak directly from the blood vessel, resulting in the formation of high-density lesions on immediate CT after the operation, and follow-up CT showed massive hemorrhage. Therefore, we use the highest CT value to predict IH, rather than the averaged CT value, and HALs mostly appear in low-density areas on preoperative CT and high-intensity areas on DWI scans.

At present, the effective method of identifying IH after ET is immediate dual source CT (18). Due to the progress made by the Chinese Stroke Center, thrombectomy has been widely promoted as a suitable treatment method for the Primary Stroke Center. However, the Primary Stroke Center is rarely equipped with dual-source CT. The method proposed in this study can use ordinary CT to predict hemorrhage. At the same time, although ducal energy CT can separate IH or iodine immediately, it does not predict hemorrhage transformation in postoperative and this approach might. Some scholars have pointed out that some patients with immediate dual source CT excluded IH had intracranial hemorrhage transformation in the follow-up CT (23.1% had delayed ICH and 11.5% had delayed PH)(15). The appearance of HALs on immediate CT can be divided into three categories. First, HALs are from hemorrhage combined with contrast agents, such patients would need blood pressure control, sedation after surgery (if necessary), and avoid the use of antithrombotic drugs. Such patients will show hemorrhage on follow-up CT. Second, HALs are from single contrast agent exudation and hemorrhage is highly likely to be transformed. The postoperative management of such patients is the same as in the first case. Third, HALs are from single contrast agent exudation and intracranial hemorrhage transformation is unlikely: these patients benefit from early antithrombotic drugs to prevent re-embolism. Thus, our study focuses on how HALs can be quantitatively analyzed to help with postoperative management, rather than be used to differentiate contrast agent exudation from IH.

This study attempts to identify patients with HALs yet with a low risk of hemorrhage. According to our results, patients with the highest CT value of <97 have a low risk of hemorrhage transformation. This value might be used to help determine patients to apply antithrombotic drugs in the early postoperative period, especially those with mTICI ≤ 2b and with emergency stent implantation or balloon dilatation who need antiplatelet to maintain the blood flow.

One limitation of this study is the relatively small sample size of the enrolled patients because only one stroke center was considered. In this case, we only generated 37 pairs of matched data, which resulted in the confidence interval ranging from 2.485 to 57.284 and partly limits the generalization of our conclusion. Moreover, more quantitative parameters within the HALs could be calculated and analyzed besides the highest CT value to predict IH. For instance, if the HALs can be lineated automatically with high repeatability and consistency, average CT value within HALs might be used.

## Conclusion

If the patients with anterior LVO are matched with the baseline characteristics of patients with perioperative IH and non-IH, the incidence of HALs on immediate CT is higher in the IH group. In all patients with HALs, the highest CT value in the HALs area might be used to predict IH in patients during the perioperative period.

## Data Availability Statement

The raw data supporting the conclusions of this article will be made available by the authors, without undue reservation.

## Ethics Statement

The studies involving human participants were reviewed and approved by Institutional Review Board at the Xi'an No.3 Hospital (No. SYXSLL-2018-010). Written informed consent for participation was not required for this study in accordance with the national legislation and the institutional requirements.

## Author Contributions

MC and YT: conception and design. NH: data collection and article draft. GC, YL, HM, HG, XZ, YZ, SL, LZ, YG, WS, PY, and WL: contributed to manuscript review and revision.

## Conflict of Interest

YL was employed by company GE Healthcare China. The remaining authors declare that the research was conducted in the absence of any commercial or financial relationships that could be construed as a potential conflict of interest.

## Publisher's Note

All claims expressed in this article are solely those of the authors and do not necessarily represent those of their affiliated organizations, or those of the publisher, the editors and the reviewers. Any product that may be evaluated in this article, or claim that may be made by its manufacturer, is not guaranteed or endorsed by the publisher.

## References

[1] Nogueira RG Jadhav AP Haussen DC Bonafe A Budzik RF Bhuva P. Thrombectomy 6 to 24 hours after stroke with a mismatch between deficit and infarct. N Engl J Med. (2018) 378:11–21. 10.1056/NEJMoa1706442 29129157

[2] Albers GW Marks MP Kemp S Christensen S Tsai JP Ortega-Gutierrez S. Thrombectomy for stroke at 6 to 16 hours with selection by perfusion imaging. N Engl J Med. (2018) 378:708–18. 10.1056/NEJMoa1713973 29364767 PMC6590673

[3] Powers WJ Rabinstein AA Ackerson T Adeoye OM Bambakidis NC Becker K. 2018 Guidelines for the early management of patients with acute ischemic stroke: a guideline for healthcare professionals from the American Heart Association/American Stroke Association. Stroke. (2018) 49:e46–e110. 10.1161/STR.0000000000000172 29367334

[4] Khatri P Wechsler LR Broderick JP. Intracranial hemorrhage associated with revascularization therapies. Stroke. (2007) 38:431–40. 10.1161/01.STR.0000254524.23708.c9 17234988

[5] Tarlov N Nien YL Zaidat OO Nguyen TN. Periprocedural management of acute ischemic stroke intervention. Neurology. (2012) 79(13 Suppl. 1):S182–S91. 10.1212/WNL.0b013e31826958d3 23008396

[6] Amaro S Llull L Urra X Obach V Cervera Á Chamorro Á. Risks and benefits of early antithrombotic therapy after thrombolytic treatment in patients with acute stroke. PLoS One. (2013) 8:e71132. 10.1371/journal.pone.0071132 23951093 PMC3738638

[7] Leslie-Mazwi T Chen M Yi J Starke RM Hussain MS Meyers PM. Post-thrombectomy management of the ELVO patient: Guidelines from the Society of NeuroInterventional Surgery. J Neurointerv Surg. (2017) 9:1258–66. 10.1136/neurintsurg-2017-013270 28963364

[8] An H Zhao W Wang J Wright JC Elmadhoun O Wu D. Contrast staining may be associated with intracerebral hemorrhage but not functional outcome in acute ischemic stroke patients treated with endovascular thrombectomy. Aging Dis. (2019) 10:784–92. 10.14336/AD.2018.0807 31440384 PMC6675522

[9] Larrue V Von Kummer RR Müller A Bluhmki E. Risk factors for severe hemorrhagic transformation in ischemic stroke patients treated with recombinant tissue plasminogen activator: a secondary analysis of the European-Australasian Acute Stroke Study (ECASS II). Stroke. (2001) 32:438–41. 10.1161/01.STR.32.2.438 11157179

[10] Renú A Amaro S Laredo C Román LS Llull L Lopez A. Relevance of blood-brain barrier disruption after endovascular treatment of ischemic stroke: dual-energy computed tomographic study. Stroke. (2015) 46:673–9. 10.1161/STROKEAHA.114.008147 25657188

[11] Rouchaud A Pistocchi S Blanc R Engrand N Bartolini B Piotin M. Predictive value of flat-panel CT for haemorrhagic transformations in patients with acute stroke treated with thrombectomy. J Neurointerv Surg. (2014) 6:139–43. 10.1136/neurintsurg-2012-010644 23468539

[12] Nikoubashman O Reich A Gindullis M Frohnhofen K Pjontek R Brockmann MA. Clinical significance of post-interventional cerebral hyperdensities after endovascular mechanical thrombectomy in acute ischaemic stroke. Neuroradiology. (2014) 56:41–50. 10.1007/s00234-013-1303-1 24306553

[13] Lee M Saver JL Alger JR Hao Q Starkman S Ali LK. Blood-brain barrier permeability derangements in posterior circulation ischemic stroke: frequency and relation to hemorrhagic transformation. J Neurol Sci. (2012) 313:142–6. 10.1016/j.jns.2011.08.048 21945462 PMC3254818

[14] Li F Yang L Yang R Xu W Chen FP Li N. Ischemic stroke in young adults of Northern China: characteristics and risk factors for recurrence. Eur Neurol. (2017) 77:115–22. 10.1159/000455093 28052272 PMC5467437

[15] Ebashi R Ogata A. Significance of simulated conventional images on dual energy CT after endovascular treatment for ischemic stroke. J Neurointerv Surg. (2019) 11:898–902. 10.1136/neurintsurg-2018-014486 30670626

[16] Khatri R McKinney AM Swenson B Janardhan V. Blood-brain barrier, reperfusion injury, and hemorrhagic transformation in acute ischemic stroke. Neurology. (2012) 79(13 Suppl. 1):S52–S7. 10.1212/WNL.0b013e3182697e70 23008413

[17] Huang S Kim JK Atochin DN Farrar CT Huang PL Suh JY. Cerebral blood volume affects blood-brain barrier integrity in an acute transient stroke model. J Cereb Blood Flow Metab. (2013) 33:898–905. 10.1038/jcbfm.2013.27 23462571 PMC3677109

[18] Almqvist H Holmin S Mazya MV. Dual energy CT after stroke thrombectomy alters assessment of hemorrhagic complications. Neurology. (2019) 93:e1068–e75. 10.1212/WNL.0000000000008093 31409735

